# Role of Extracellular Vesicles in Epithelial Ovarian Cancer: A Systematic Review

**DOI:** 10.3390/ijms21228762

**Published:** 2020-11-19

**Authors:** Alessandro Lucidi, Danilo Buca, Carlo Ronsini, Sara Tinari, Giuseppina Bologna, Davide Buca, Martina Leombroni, Marco Liberati, Francesco D’Antonio, Giovanni Scambia, Paola Lanuti, Marco Petrillo

**Affiliations:** 1 Obstetrics and Gynecology Clinic, University of Chieti, 66100 Chieti, Italy; lucidi.alex@gmail.com (A.L.); danilobuca@gmail.com (D.B.); carlo.ronsini90@gmail.com (C.R.); satynari@gmail.com (S.T.); martina.leombroni@gmail.com (M.L.); marco.liberati@unich.it (M.L.); dantoniofra@gmail.com (F.D.); 2 Department of Medicine and Aging Sciences, University “G. d’Annunzio”, Chieti—Pescara, 66100 Chieti, Italy; giuseppina.bologna@hotmail.it (G.B.); bucadavide@gmail.com (D.B.); 3 Center for Advanced Studies and Technology (C.A.S.T.), University “G. d’Annunzio”, Chieti-Pescara, 66100 Chieti, Italy; 4 Department of Woman and Child Health, IRCCS Fondazione Policlinico Agostino Gemelli, 00168 Rome, Italy; giovanni.scambia@policlinicogemelli.it; 5 Department of Medical, Gynecologic and Obstetric Unit, University of Sassari, 07100 Sassari, Italy; marco.petrillo@gmail.com

**Keywords:** extracellular vesicles, tumor microenvironment, ovarian cancer, microRNAs, epithelial mesenchymal transition, immunological escape

## Abstract

Extracellular vesicles (EVs) are a heterogeneous group of cell-derived submicron vesicles released under physiological or pathological conditions. EVs mediate the cellular crosstalk, thus contributing to defining the tumor microenvironment, including in epithelial ovarian cancer (EOC). The available literature investigating the role of EVs in EOC has been reviewed following PRISMA guidelines, focusing on the role of EVs in early disease diagnosis, metastatic spread, and the development of chemoresistance in EOC. Data were identified from searches of Medline, Current Contents, PubMed, and from references in relevant articles from 2010 to 1 April 2020. The research yielded 194 results. Of these, a total of 36 papers, 9 reviews, and 27 original types of research were retained and analyzed. The literature findings demonstrate that a panel of EV-derived circulating miRNAs may be useful for early diagnosis of EOC. Furthermore, it appears clear that EVs are involved in mediating two crucial processes for metastatic and chemoresistance development: the epithelial–mesenchymal transition, and tumor escape from the immune system response. Further studies, more focused on in vivo evidence, are urgently needed to clarify the role of EV assessment in the clinical management of EOC patients.

## 1. Introduction

Despite the efforts made in recent decades to improve the efficacy of therapeutic strategies, epithelial ovarian cancer (EOC) remains the most lethal among gynecological malignancies. From a clinical point of view, the cornerstones in the management of EOC are still represented by a combination of surgery and chemotherapy. However, the development of innovative and more effective surgical and medical approaches was associated with only a slight survival improvement [1,2]. The unfavorable prognosis of OC patients is mainly due to late stage diagnosis, and the development of chemoresistance along disease natural history [3,4,5,6].

Focusing on diagnosis, OC remains an asymptomatic disease up to the occurrence of diffuse peritoneal carcinomatosis, which includes abdominal bloating, and gastrointestinal symptoms [1]. Furthermore, the combination of trans-vaginal ultrasonography and dosage of CA-125 serum levels, as screening methods, demonstrated a promising, but still limited, ability to identify EOC as early stage disease [1,2]. As a consequence, in approximately 70% of patients, EOC is diagnosed as late stage disease with a 5-year overall survival below 40% [4,5,6], thus emphasizing the need to develop highly sensitive, non-invasive tools able to ensure an early diagnosis, addressing one of the biggest challenges in biomarker research.

As previously highlighted, another crucial point in EOC research is certainly represented by the development of drug resistance [6,7,8]. Relevant efforts have been pointed out in identifying biological factors able to predict the development of platinum resistance, and eventually suitable as therapeutic targets to increase response to platinum compounds.

In this context, growing attention has been focused on the role of extracellular vesicles (EVs) and their cargoes as prognostic and predictive factors in EOC patients. In fact, these micro-environmental structures are involved in cellular crosstalk, contributing to several physiological responses, such as metabolism regulation and activation of stress response, but, at the same time, EVs may represent an effective tool for cancer tissue to maintain its own pathological homeostasis [6,7,8].

For these reasons, we review here the available literature investigating the role of EVs in OC patients, trying to emphasize the clinical implication in the diagnosis and treatment of this challenging malignancy.

## 2. Search Strategy and Selection Criteria

The design of this systematic review of the literature followed PRISMA guidelines. Data were identified from searches of Medline, Current Contents, PubMed, and from references in relevant articles published from January 2010 to April 2020, using the following search terms: “ovarian cancer”, “early-stage ovarian cancer”, “high grade ovarian cancer”, “exosome”, and “ovary”.

The research yielded 194 results. Only articles published in English were included.

We identified a total of 29 papers, 5 reviews, and 24 original research, and these data were retrieved and analyzed (Table S1).

## 3. Extracellular Vesicles Subtypes and Methods of Analysis

### 3.1. Extracellular Vesicles: Definition and Classification

Extracellular vesicles (EVs) are a heterogeneous group of cell-derived submicron vesicles released under physiological or pathological conditions [9,10,11,12,13,14,15,16,17]. In the past decade, EVs have been classified on the basis of their size and mechanisms of biogenesis, as exosomes (30–100 nm), when generated within endosomal compartments, microvesicles (MVs, 100–1000 nm), and apoptotic bodies (0.1–5 µm), both budding directly from the plasma membrane [18].

- Exosomes, the smallest EVs, originate within the lumen of multivesicular bodies (MVBs), and their release process is regulated by a group of proteins called “endosomal sorting complex required for transport” (ESCRT) or through ESCRT-independent mechanisms [19,20]. In any case, when MVB vesicles fuse with the plasma membrane, they release their content in the extracellular milieu as exosomes. It has been demonstrated, by immunoelectron microscopic studies, that exosomes are enriched in tetraspanins that seem to play a key role in the exosome release as well as in exosomal sorting of cargo molecules [21,22,23,24,25,26,27]. For these reasons, tetraspanins CD9, CD63, and CD81 were pointed out as specific exosome markers [28].
- MVs, released by budding from their parental cells, include a subtype of EVs released by cancer cells, well known as “oncosomes”. The presence of larger oncosomes (1–10 µm in diameter) has been also described, with the involvement of these EVs in driving metastatic spread [29,30] through integrin signaling regulation [29].
- Apoptotic bodies, released by cells undergoing apoptosis, can be distinguished from other phosphatidylserine-positive MVs based on positivity for caspases 3 and 7 and their substrates [31].

It should be also mentioned that several cell types, and resting platelets, secrete respiratory-competent mitochondria susceptible to autonomous extracellular signaling as well as to intercellular transfer, a finding whose implications are still unclear but that could widely expand the scope of cell–cell communication biology [32]. To conclude, classifications mainly based on size do not fit the heterogeneity of the EV populations and their overlaps in cargo, biodistribution, and functions [33]. Thus, in a recent position paper, the International Society of Extracellular Vesicles (ISEV) endorsed the use of the term “extracellular vesicle” for all EV types, with a generic EV subclassification as small, if within 100 nm, and medium/large, if above 100–200 nm [29]. Finally, it must be underlined that despite relevant efforts made in order to establish specific phenotypes identifying the different EV subtypes, the reliability of this classification system was not satisfying, thus leading the ISEV to establish that it is not possible to classify each EV type through specific markers [29].

### 3.2. Extracellular Vesicles: Methods of Identification and Analysis

Several methods were used to identify and analyze EVs [29,34]. First, EV-specific proteins (e.g., specific tetraspanins) have been identified and are frequently detected by immunoblotting to confirm the presence of EVs in biological samples [29,35]. Transmission electron microscopy (TEM), scanning EM (SEM), and cryogenic TEM are largely applied to determine EV features (diameters and morphology) [36,37,38,39,40,41], while specific physical EV properties, such as EV stiffness and elasticity, can be measured by atomic force microscopy (AFM) [42,43,44]. EV size distribution and polydispersity in a sample can be also analyzed by dynamic light scattering (DLS), which detects the diffusion coefficient of the scattering EVs [45]. Nanoparticle tracking analysis (NTA) allows the detection of both EV dimensions and concentrations, through the analysis of the EV Brownian motion and the measurement of scattered light (Sc-NTA) or emitted fluorescence (Fl-NTA) [46]. Tuneable resistive pulse sensing (TRPS) measures changes in the electrical current as each EV passes through an adjustable nanopore [47,48]. Asymmetric flow field-flow fractionation (AF4) separates EVs on the basis of their hydrodynamic size, making it possible to identify even the smallest compartment of EVs (a few nanometers in diameter) [49]. However, it must be underlined that flow cytometry (FC) is currently considered as the most appropriate technique for EV analysis [50,51,52,53]. The polychromatic FC analysis, using tracers that stain the whole compartment of intact EVs (i.e., lipophilic carbocyanine dyes, combined with phalloidin, that selectively bind to F-actin), accurately discriminates EVs from debris and artefacts [13,54,55,56,57]. Polychromatic FC also allows EV immunophenotypic characterization through the combination of specific antibodies [13,28,34,58,59]. Imaging flow cytometers combining conventional FC with fluorescence images provide a new sensitive tool for EV studies [60,61].

### 3.3. Extracellular Vesicles: Biological Role

From a functional point of view, it must be underlined that EVs play relevant roles in intercellular communication, being involved in several physio-pathological activities such as coagulation, angiogenesis, cell survival, cancer progression, modulation of the immune response, and inflammation, as well as in fetal–maternal communication [10,15,62,63]. It has been demonstrated that cancer cells show an increased EV production, when compared with normal cells, and this could be related to the specific conditions of the tumor microenvironment [64]. As critical mediators of cell-to-cell communication, EVs may play an important role in cancer progression, by mediating the crosstalk between tumor and stromal cells in their own environment. In addition, circulating EVs carry complex biological information from their donor cells and should have an emerging role as a new type of cancer biomarker [65,66,67].

## 4. Extracellular Vesicles and OC: Focus on Diagnosis

### 4.1. Background

Several molecular factors have been investigated as potential biomarkers in epithelial ovarian cancer (EOC), given their role in driving the mechanisms of oncogenesis, tumor invasion, and metastatic spread. In the last decade, growing attention has been dedicated to the potential role of single-stranded non-coding RNAs, that are typically 19–25 nucleotides in length, best known as microRNAs (miRNAs). It is well established that miRNAs are endogenously expressed key regulators of post-transcriptional gene expression [68]. The vast majority of miRNAs circulate in the body fluids of patients as cell-free RNAs; however, in the group of cell-free RNAs, those packaged in EVs (microvesicles, exosomes, or apoptotic bodies) are the most stable, thus being more effective in driving the process of intercellular crosstalk. Many EV-embedded circulating miRNAs are hyper- or hypoexpressed in EOC patients compared to healthy women, and so they may be considered as potential innovative biomarkers to be used in clinical practice [69]. In particular, the detection of blood-circulating miRNAs located in EVs is currently known as liquid biopsy, a minimally invasive promising blood-based approach that has the potential of providing information on prognosis, response to therapeutic regimens, early diagnosis, and population screening [70]. Besides miRNAs, EVs also contain proteins located on the surface or embedded in the microvesicles, which may also retain a potential role as diagnostic biomarkers. In this context, EVs emerge as a novel interesting research scenario, since a panel of EV-related miRNAs and proteins may act in future as a “cancer signature”, representing a useful new weapon in EOC screening and diagnosis.

### 4.2. Literature Data

This promising scenario seems to be supported by relevant evidence from the literature (Table S1), as summarized below.

In 2019, Barnabas et al. found several biomarkers overexpressed in utero-tubal lavage (UtL) of 49 women with high-grade serous ovarian cancer (HGSOC) compared to 121 healthy women [70]. In particular, nine EV-related proteins were differentially expressed between the two groups: SERPINB5 (an epithelial cell-specific member of the SERPIN family that lacks serine protease inhibition activity), S100A14 (a member of the S100 family lacking calcium-binding function, known to be involved in the regulation of TP53 protein expression and of cellular motility), MYH11, CLCA4, S100A2, IVL, CD109, NNMT, and ENPP3. The dosage of this nine-protein panel showed interesting diagnostic performance in the early diagnosis of HGSOC, with a sensitivity and specificity of 70% and 76.2%, respectively.

Focusing on EV-related miRNAs, Chi et al. in 2018 showed a statistically significant overexpression of miR-23a, miR-92a, miR-21, miR-100, and miR-200b in exosomes from the plasma of EOC patients when compared with healthy women [71]. At the same time, the authors observed reduced expression of miR-320, miR-16, miR-93, miR-126, and miR-223. In particular, miR-21 showed a sensitivity of 61% and a specificity of 82% in discriminating between EOC patients and healthy women. At the same time, miR-100 and miR-200b presented a sensitivity of 62% and 64%, respectively, with a specificity of 73% and 86%. Unfortunately, the combination of those values did not increase the related detection power. In 2018, Cindy et al. demonstrated a hyperexpression of NANOG; SPINT2; ZEB2 in EOC patients [2]. Furthermore, NANOG; SPINT2; ZEB2 were found to be significantly overexpressed in ascitic fluids of eight EOC patients compared with peritoneal fluids of 10 healthy patients. On the other hand, miR29a; miR30d; miR205; CA11; LAMA4 and MEDAG have been proved to be hypoexpressed in ascites samples. A combination of LAMA4, CA11, MEDAG, NANOG, SPINT2, let-7b, miR23b, and miR29a was finally investigated, showing promising diagnostic performances, but the small sample size does not allow us to draw reliable conclusions.

In the same year, Akihiko Yoshimura et al. showed an overexpression of miR-99a-5p in the serum of 62 OC patients compared with 20 healthy controls [72]. It also significantly decreases after debulking surgery (*p* = 0.003), thus showing a potential correlation with tumor burden. The authors established a cut-off value for miR-99a-5p expression levels and, using this threshold, the diagnostic performances for detecting EOC were promising, with a sensitivity of 85%, and a specificity of 75%. Interestingly, the abovementioned data were significantly different according to tumor histology (sensitivity and specificity: 84% and 40%, for serous EOC; 33% and 91%, for clear-cell EOC; 67% and 82%, for endometrioid EOC; 33% and 91%, for mucinous EOC), which also strongly suggests the need for tailored approaches in diagnostic evaluations. Finally, when compared with the CA125 dosage, miR-99a-5p showed an improved sensitivity (87 vs. 54), with a comparable specificity (73 vs. 75) in the early detection of EOC, thus deserving further investigations on its clinical role.

Wang et al. demonstrated that five miRNAs in plasma exosomes (miR-205-5p, miR-145-5p, miR-10a-5p, miR-346, and miR-328-3p) were significantly overexpressed in patients with EOC (32 women) when compared with a control group (32 healthy women) [62]. When those five miRNAs were combined, the diagnostic accuracy was higher (AUC: 0.760; 95% CI: 0.691–0.828) compared with the dosage of one miRNA alone. 

Masaki Kobayashi et al. [73] in 2018 demonstrated that miR-1290 was highly expressed in exosomes detectable in sera of 70 EOC patients. Among these, 72% of women showed advanced stage (FIGO stage III/IV) and 27% early stage (FIGO stage I/II) disease, but, when these data were compared with healthy controls (13 patients), no statistically significant difference was observed (*p* = 0.89).

In 2017, Reiner et al. showed a hyperexpression of cellular fibronectin (FN1-EDA), protectin (CD59), epithelial cell adhesion molecule (EpCAM), β-catenin, and the gelatinases matrix metalloproteinase 2 and 9 (MMP2/9) in EOC patients, compared with healthy women [74]. FN1-EDA, also called cellular fibronectin, is a splice variant of fibronectin and a crucial component of the extracellular matrix. It is highly expressed during embryogenic development, but barely found in adult tissues in contrast to the soluble fibronectin isotype, which is constantly produced by hepatocytes and generally present in plasma [75]. FN1-EDA was detected in the exosomes in the ascites of EOC patients.

In this context, special attention needs to be focused on EpCAM serum levels. After the results published by Reiner et al. [74], the role of EpCAM as promising biomarker was also confirmed in the study published by Zhang et al. [76], in which the authors performed a quantitative proteomic analysis (iTRAQ) of circulating EVs in a group of 10 EOC patients, and 10 healthy controls. Proteomic analysis revealed that 200 proteins were upregulated and 208 proteins were downregulated in the EOC group. The most interesting results are represented by the overexpression of four serum EV proteins: EpCAM, C1q, ApoE, and plasminogen, with a downregulation of serpin C1. ApoE multiplexed with EpCAM, Plg, serpin C1, and C1q provides optimal diagnostic performance for EOC with an impressive area under the curve (AUC ) of 0.913 (95% confidence interval (CI) = 0.848–0.957, *p* < 0.0001). However, these very promising data must be validated in larger study groups prior to being considered in clinical practice.

In 2012, Liang et al. demonstrated that EOC-derived exosomes carried a protein expression signature that was also overexpressed in EOC tissues, including epithelial cell adhesion molecule (EpCAM), proliferation cell nuclear antigen (PCNA), tubulin beta-3 chain (TUBB3), epidermal growth factor receptor (EGFR), apolipoprotein E (ApoE), claudin 3 (CLDN3), fatty acid synthase (FASN), ERBB2, and L1 cell adhesion molecule (L1CAM), suggesting that these proteins could be used as diagnostic markers for OC [77].

In 2017, Stope et al. [78] found a high in vitro expression of heat shock protein 27 (HSP27) in the OVCAR-3 cell line (1004.9 ± 137.4 pg/mL). HSP27 directly interacts with other oncoproteins to regulate cell survival. As is well known, increased levels of HSP27 in tumor cells are due to the loss of p53 functions along with higher expression of proto-oncogenes; furthermore, HSP27 appears to play a role in angiogenesis and inflammation [77,79]. The authors also demonstrated increased HSP27 levels in EVs of EOC patients and, following this evidence, HSP27 has been proposed as a potential biomarker of peritoneal carcinomatosis in women with EOC [80]. In fact, HSP27 serum levels in patients with peritoneal metastasis (23 patients) were significantly higher than those in patients without peritoneal metastasis (25 patients) (5.475 ng/mL vs. 3.276 ng/mL, *p* = 0.032,) or in healthy women (2.46 ng/mL, *p* = 0.005). Therefore, EV-derived HSP27 appears as a potentially useful biomarker, which deserves further investigations as a promising tool in liquid biopsies to achieve early diagnosis of EOC.

### 4.3. Take Home Message

In conclusion, EV-derived miRNAs and proteins appear as a promising biomarker to achieve the challenging goal of improving early detection of EOC. The critical evaluation of the published studies strongly suggests that a panel of EV biomarkers may be more useful than a single one. However, even if literature data appear promising, several limitations do not allow us to draw definitive conclusions, including: the small sample size of investigated cohorts of patients, with no external validation, and the lack of studies evaluating the role of the mentioned biomarkers when integrated in clinical algorithms (including symptom evaluation, CA125 dosage, and ultrasound findings). The future horizon is certainly represented by addressing these issues proposing, in the clinical scenario, the most reliable panel of EV biomarkers.

## 5. Extracellular Vesicles and OC: Focus on Prognosis

### 5.1. Literature Data

In 2018, Zhou et al. analyzed by immunofluorescence techniques the ovarian tissue samples from 124 EOC patients and 26 women with benign ovarian tumors in order to identify the prognostic relevance of Treg and Th17 infiltration in tumor tissues [3]. Interestingly, the authors showed a statistically significant higher Treg/Th17 ratio in EOC patients’ tumor tissues, blood samples, and ascitic fluids compared to women with benign ovarian disease (*p* < 0.001) [4]. Furthermore, the authors observed that EVs released from tumor-associated macrophages (TAMs, type M2) induce an imbalance in Treg/Th17 cells, favoring Treg differentiation. The pathway which promotes these mechanisms is linked with an exosomal overexpression of hsa-miR-21-5p (*p* = 0.001); hsa-miR-24-3p (*p* = 0.0037); hsa-miR-29a-3p (*p* = 0.037); hsa-miR-146b-5p (*p* = 0.037) and hsa-miR- 660-5p (*p* = 0.032). These miRNAs could bind to the STAT3 3′ UTR, which in turn promotes CD4+ T cell differentiation into Treg or Th17 cells. Focusing on prognostic data, the authors analyzed a cohort of women retrieved from The Cancer Genome Atlas (TCGA), showing statistically significant shorter overall survival (OS) in EOC patients with a higher Treg/Th17 ratio (40 vs. 51 months, *p* = 0.043); a similar trend toward a reduced OS was also observed in women showing increased expression levels of EV miR-29a-3p and miR-21-5p. Furthermore, an increased Treg/Th17 ratio was also associated with poorer histological grade of differentiation.

A study published in 2017 (1582 patients) by Yokoi et al. [5] found that EVs carrying MMP1 mRNA (matrix metalloproteinase 1 mRNA) are involved in peritoneal dissemination. The ascites-derived EVs carrying MMP1 miRNAs were responsible for a destructive phenotype in mesothelial cells, inducing their apoptosis and leading to the beginning of peritoneal dissemination. MMP1 expression levels could be a potential biomarker of poor prognosis (hazard ratio (HR), 1.24; log-rank test, *p* = 0.013). This topic has been recently addressed in a literature review by Nakamura et al. [6], in which several exosome-derived molecules were identified as involved in invasion, migration, and metastatic spread and therefore identified as unfavorable prognostic biomarkers in malignant ascites, including: STAT3/Fas; epithelial cell adhesion molecule (EpCAM); membrane-type 1, 2, and 9 MMP (MMP-1; MMP-2; MMP-9); urokinase-type plasminogen activator; and soluble L1 adhesion molecule (CD171). Shimizu et al. [80,81] paid particular attention to the relationship between cancer-derived exosomes and the cancer microenvironment. Indeed, ovarian cancer cell-derived exosomes convert fibroblasts into cancer-associated fibroblasts (CAFs). Thereafter, CAF-derived exosomes promote the epithelial–mesenchymal transition to ovarian cancer cells through the production of transforming growth factor 1 (TGFβ 1), that in turn activates SMAD signaling. Moreover, cancer-derived exosomes produce activating transcription factor 2 (ATF2), metastasis-associated protein 1(MTA1), and CD147, all angiogenic factors, in response to the hypoxic noxa given by tumor growth, and the associated increased metabolic demand. Additionally, metastasis-associated lung adenocarcinoma transcript 1 (MALAT1) performs the same function. It has also been recently demonstrated that the hyperexpression of MALAT1 may play a role in several malignancies, including ovarian, cervical, and breast cancer [82,83,84]. In particular, MALAT1 is a long non-coding RNA, which promotes angiogenesis and metastatic spread in EOC, being transferred through EVs from cancer cells to recipient healthy cells, where it acts by enhancing angiogenesis-related gene expression. Additionally, elevated serum levels of EV-derived MALAT1 have been correlated with advanced and metastatic stages of disease, thus being suitable as a prognostic factor in EOC patients [84]. In 2017, Lutgendorf et al. reported the presence of a difference between plasma-derived exosomes from EOC patients with low and high social support (more or less than USD 80,000 annual income) [84]. Specifically, exosomes from patients with low social support showed increased expression of epithelia-mesenchymal transition (EMT)-related gene transcripts in comparison with their counterparts with high social support, and this reflected the increased expression of a composite of mesenchymal genes, but no difference in the expression of the epithelial gene composite. These data support the possibility of using exosomes as a non-invasive liquid biopsy to assess the effects of bio-behavioral factors and help to direct personalized treatment approaches for cancer that optimize patient-level physiological influences on cancer biology.

### 5.2. Take Home Message

Despite the high lethality of EOC, only few prognostic biomarkers have successfully entered clinical practice (CA125, BRCA1/2, etc.). The available literature strongly supports the role of several EV-derived miRNAs and protein as being involved in driving the process of EMT and metastatic development, thus suggesting that in the future, a panel of these biological factors may be used to achieve a more accurate prognostic characterization of EOC patients.

## 6. Extracellular Vesicles and OC: Therapeutic Issues

### 6.1. Actionable Therapeutic Targets

EOC is currently treated with a combination of cytoreductive surgery and adjuvant or neoadjuvant platinum/taxane-based chemotherapy [7,85,86,87]. Furthermore, in recent years, the inhibitors of poly (ADP-ribose) polymerases (PARPis) emerged as an exciting therapeutic option and demonstrated an outstanding activity in EOC, and is currently changing clinical practice in BRCA mutant and high-grade serous ECO patients [7,85,86,87]. This relevant achievement is attributable to the increased knowledge of the biological mechanisms sustaining OC development. In this landscape, miRNAs are emerging as actionable targets for novel therapies, given their role in driving angiogenesis, invasion, metastasis, chemoresistance, and immunological escape. In this new therapeutic frontier, several EVs have been recognized as key regulators of tumor progression, given their ability to suppress system response, and in particular T cell activation.

In 2019, Feng et al. [7] summarized the pathogenetic issue of EVs released by primary ovarian malignancies. In fact, EOC-derived exosomes promote pre-metastatic niche formation via immunosuppression, angiogenesis, stromal cell remodeling, and oncogenic reprogramming. In particular, miR-31 and miR-214 downregulation with miR-155 upregulation promote the transformation of fibroblasts in cancer-associated fibroblasts (CAFs), which are unique, reprogrammed stromal cells with roles in cancer initiation, extracellular matrix remodeling, pre-metastatic niche formation, and metastatic spread [85]. In the pre-metastatic niche, the tumor-derived exosomes convert local fibroblasts into CAFs, which support tumorigenesis through their exosome secretion. When ovarian cancer cells take up TGFβ1-enriched CAF exosomes, they upregulate TGFβ1 expression, activating migration and invasion through an SMAD signaling cascade [86]. CAFs also enhance basement membrane permeability, allowing tumor cells to better invade the local uninvolved stroma. Ovarian cancer-associated exosomes can also induce the production of IL-6 within monocytes through Toll-like receptor (TLR) activation. IL-6 then activates the signal transducer and activator of transcription 3 (STAT3) pathway in immune cells, stromal cells, and tumor cells, which supports the overall immune escape of cancer cells. In addition to reprogramming the immune cell gene profile, ovarian cancer cells release Fas ligand (FasL)-carrying exosomes which downregulate the expression of the surface T cell receptors/CD3-zeta (ζ) and promote T cell apoptosis [87]. Furthermore, proteomic analysis revealed metastasis-associated protein 1 (MTA1), activating transcription factor 2 (ATF2), and E-cadherin (sE-cad) housed within ovarian cancer exosomes as being involved in upregulating angiogenesis [88]. Finally, ovarian cancer cells release exosomes with specific miRNAs (miR-21 and miR-29a) into the ascites that remodel the mesothelial cell layer, thus enhancing peritoneal penetration [88]. Therefore, these EV-derived miRNAs appear as actionable targets for tailored therapies, which could in principle selectively interrupt the carcinogenesis pathways [89].

In 2011, Peng Peng et al. [8] reported that exosomes derived from ascites of EOC patients express immunological cell surface antigens, which means that ascites-derived EVs are released by immunological cells [8]. In particular, Nakamura et al. clearly summarized evidence suggesting that phosphatidylserine and FasL derived from EVs are responsible for the inhibition of T cell activity and T cell apoptosis, being increased in ascites of EOC patients [6]. Taken together, these results strongly suggest that EVs are involved in mediating the apoptotic processes in EOC tissues, being therefore suitable for future anticancer therapies. More recently, Kuzmicz et al. demonstrated that EVs obtained from the plasma of patients with EOC contained more arginase 1 (ARG-1) than the control group [90], with an increased plasma arginase activity. It should be noted that ARG-1 is a well-known inhibitor of T cell function in cancer patients, thus suggesting other mechanisms of EV-mediated immune suppression in women with EOC. Furthermore, the authors demonstrated a reduced proliferation of peripheral blood CD4+ and CD8+ T cells when incubated with EOC ascitic fluid-isolated EVs compared to benign cyst fluid-isolated EVs.

In 2018, Xin Chen et al. showed that SKOV3 cells lines, after a hypoxic shock, synthesize miR-21-3p; miR-125b-5p; miR-181d-5p [91]. These three miRNAs were involved in activating the SOCS/STAT3 pathway. In particular, the related western blot analysis demonstrated that, compared with the normal cell line, SKOV3 transfection with miR-21-3p, miR-125b-5p, and miR-181d-5p increased the phosphorylation of STAT3. p-STAT3 acts in turn induces M2 polarization of tumor-associated macrophages, which ultimately contribute to the development of the metastatic phenotype of EOC cells [92]. This evidence further confirms that EVs derived from EOC patients are deeply involved in promoting the cancer-related immune-tolerogenic milieu, opening the horizon for future tailored therapeutic strategies.

It must be underlined that, in adult life, epithelial–mesenchymal transition (EMT) may occur during cancer development. The intercellular communication between stem cells, stromal cells, and epithelial cells is a key event for the induction of EMT reprogramming and the acquisition of a mesenchymal stem cell (MSC) phenotype. Interestingly, EVs have been demonstrated to be EMT inducers for target cells, which are capable of reprogramming recipient cells [93]. In 2015, Enriquez et al. demonstrated that in vitro human embryonic kidney 293 cells (HEK293) overexpress genes driving EMT when exposed to LIN28A-containing EVs secreted by ovarian cancer cell lines (IGROV1) [94]. EV exposition resulted in significantly increased expression of 45 EMT-related genes, including ZEB1, NOTCH1, WNT5A, NODAL, and SNAI2. LIN28A and miRNAs can reprogram cells and are well-known regulators of cell differentiation, and these data further confirm that this type of EV could be used as a target to block tumor spread [95].

Hu et al. demonstrated that exosomes derived from tumor necrosis factor (TNF)-like weak inducer of apoptosis (TWEAK) were able to inhibit the metastatic spread of EOC cells through the shuttling of miR-7. In particular, TWEAK is a regulator of immune response and mediates an anti-tumor effect through the exosomal transfer from macrophages [96]. Furthermore, TWEAK stimulation increased miRNA-7 exosomal secretion, that consequently inhibits ovarian cancer metastasis via EGFR/AKT/ERK1/2 pathway suppression. In conclusion, the exosome-derived TWEAK/miR-7 pathway represents a promising therapeutic target.

**Figure 1:**
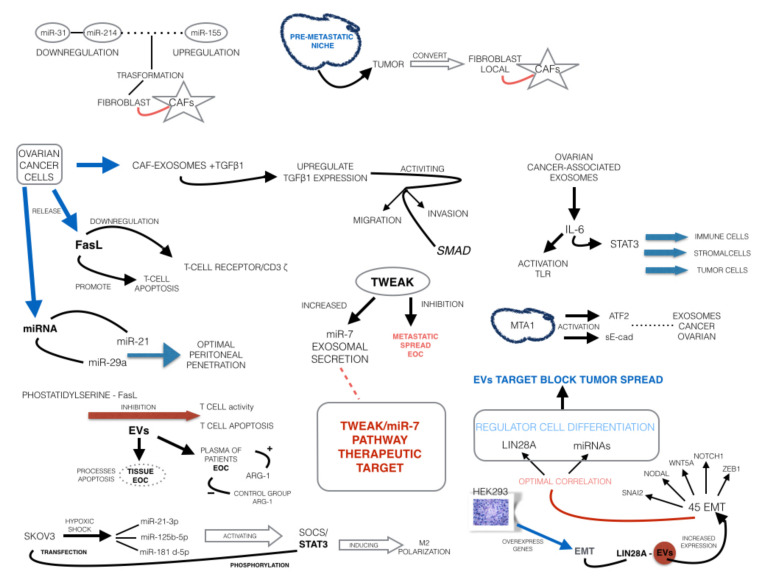
Extracellular vesicles and miRNAs as therapeutic targets in epithelial ovarian cancer (EOC).

These mechanisms are summarized in Figure 1.

### 6.2. EVs and Platinum Resistance

Platinum resistance mechanisms deserve a particular focus. Regardless of the initial response to treatment, resistance to platinum compounds develops along the disease natural history in around 20% of EOC patients, being one of the most relevant clinical challenges. Nowadays, it is not possible to predict which patients will develop platinum resistance, but accumulating evidence supports the role of EVs in these biological mechanisms [97].

In 2011, Yin et al. highlighted that patients with resistance to platinum-based chemotherapy (30 patients) show high levels of annexin A3 in sera when compared with drug-sensitive controls (20 patients) [98]. Annexin A3 is a phospholipid-binding protein involved in cellular processes such as vesicle trafficking and exocytosis. The authors demonstrated that annexin A3 promotes the formation of multivesicular bodies that are secreted with exosomes, allowing the development of drug resistance. In conclusion, serum annexin A3 levels may be a prognostic biomarker of resistance to platinum-based chemotherapy in EOC patients with reasonable sensitivity (63%) and specificity (80%).

Another protein, called RAB7A, was identified by Guerra et al. [99] as a potential biomarker of cisplatin resistance. This molecule is a key regulator of the late endocytic pathway, with a role relevant in controlling early and late endosome maturation, lysosomal biogenesis, and acidification, and also promoting the clustering and fusion of late endosomes and lysosomes in the perinuclear region. It has been also demonstrated that downregulation of RAB7A protein in chemoresistant cells could determine alterations in drug sequestration, resulting in increased drug efflux [99].

In 2018, Kuhlmann et al. [100] analyzed plasma levels of exosomal miRNAs in 30 EOC patients, showing a reduced expression of the following miRNAs, compared to healthy controls: hsa-miR-128-3p; hsa-miR- 99a-5p; hsa-let-7i-5p; hsa-miR-148a-3p; hsa-miR-129-5p; hsa-miR-381-3p; hsa-miR-9-3p; hsa-miR-9-5p; hsa-miR-433-3p; hsa-let-7b-5p; miR-181a; miR-1908; miR-486; and miR- 223. Furthermore, the authors suggest for the first time that the following miRNAs, which have been previously associated with platinum resistance in cell culture and in primary ovarian cancer tissue [101,102], could be relevant as blood-based biomarkers of platinum resistance: miR-181a, miR-1908, miR-21, miR-486, and miR-223.

## 7. Conclusions

In the last decade, experimental achievements have clearly demonstrated that the microenvironment also plays a crucial role in driving tumor development, and metastatic spread, in EOC. In this context, it is well known that EVs, with their molecular cargo, mediate the intercellular crosstalk, thus being a relevant tool to maintain the homeostasis of cancer tissue. Focusing on EOC, the results of our literature review demonstrate that a panel of EV-derived circulating miRNAs may be useful in the following years for early diagnosis, and disease monitoring during cancer therapy. Furthermore, it appears clear that EVs are involved in mediating two crucial processes for metastatic development: the epithelial–mesenchymal transition, and tumor escape from immune system response. Finally, growing evidence strongly suggests that molecules carried by EVs promote the development of resistance to platinum compounds in EOC tissue, favoring an adaptative pathologic response of cancer cells to drug exposure. In conclusion, taken together, the reviewed evidence confirms that targeting EV crosstalk in EOC tissue is a novel promising therapeutic strategy. Further studies, more focused on in vivo evidence, are urgently needed to clarify the role of EV assessment in the clinical management of EOC patients.

## Supplementary Materials

The following are available online at https://www.mdpi.com/1422-0067/21/22/8762/s1, Figure S1: Extracellular vesicles and miRNAs as therapeutic targets in epithelial ovarian cancer (EOC), Table S1: Established roles of EVs in Ovarian Cancer.
