# Supplementary material for: Role of Extracellular Vesicles in Epithelial Ovarian Cancer: A Systematic Review
Source: Int J Mol Sci. 2020 Nov 19;21(22):8762. doi: 10.3390/ijms21228762 (PMC7699467; doi:10.3390/ijms21228762)
Supplement: Supplementary file 1 [file ijms-21-08762-s001.zip › ijms-1002355-supplementary.docx]

| **Supplementary Table 1**. Established roles of EVs in Ovarian Cancer. | | | | | | | | | | |
| --- | --- | --- | --- | --- | --- | --- | --- | --- | --- | --- |
| N° | Author | Year | Study Design | Markers described | Upregulated | Downregulated | Subtypes of EVs Described | Role | Implication | Potential Usefulness |
| 1 | Susan K. Lutgendorf [84] | 2017 | Original research | 130 gene | NS | NS | Exosome | Increase CREB/ ATF and NF-jB/Rel activity; Derease Glucocorticoide receptro's activity | Prognosis | NS |
| 2 | Wei Zhang [76] | 2015 | Original research | NS | NS | NS | Exosome | Different expression in tumor | Diagnosis | NS |
| 3 | Koji Nakamura [6] | 2016 | Review | Dicer and Drosha; miR-183-3P; miR- 15b-3p; miR-15b; miR-590-5p; miR-18a; miR-16; miR-96; miR-18b; miR- 140-3p; miR-145-3p; miR-143-5p; miR-34b-5p; miR-145; miR-139-5p; miR-34c-3p; miR-133a; miR-34c-5p; miR-199a-3p; miR-21; miR-141; miR-200a; miR-200b; miR-200c; miR-203; miR-205; miR-214; miR-132; miR- 26a; let-7b; miR-145; miR-143 | miR-183-3P; miR- 15b-3p; miR-15b; miR-590-5p; miR-18a; miR-16; miR-96; miR-18b; miR-21; miR-141; miR-200a; miR-200b; miR-200c; miR-203; miR-205; miR-214 | miR- 140-3p; miR-145-3p; miR-143-5p; miR-34b-5p; miR-145; miR-139-5p; miR-34c-3p; miR-133a; miR-34c-5p; miR-199a-3p; miR-132; miR- 26a; let-7b; miR-145; miR-143 | cfRNA | Angiogenesis, invasion, and metastasis, poor surgical outcome | Diagnosis; Prognosis; Therapy | Liquid biopsy; Prognosis factor; Target therapy |
| 4 | Cindy M. Yamamoto [2] | 2018 | Original research | EpCAM; NANOG; SPINT2; ZEB2; let-7b; miR23b; miR29a; miR30d; miR205; miR720; CA11; LAMA4; MEDAG | NANOG; SPINT2; ZEB2; | miR29a; miR30d; miR205; CA11; LAMA4; MEDAG | Exosome; MV | NS | Diagnosis | Liquid biopsy |
| 5 | Agnes T. Reiner [74] | 2017 | Original research | CD9; CD63; CD71; FN1-EDA; CD59; EpCAM; MMP2/9; β-catenin; CD59 | CD9; CD63; CD71; FN1-EDA; CD59; EpCAM; MMP2/9; β-catenin; CD59 |  | Exosome | NS | Diagnosis | Liquid biopsy |
| 6 | Jun-Jun Qiu [82] | 2018 | Original research | MALAT1 | MALAT1 |  | Exosome | Angiogenesis, invasion, and metastasis | Diagnosis | prognostic factor |
| 7 | Chi Pan [71] | 2018 | Original research | miR-21; miR-100; miR-200b; miR-320; miR-16; miR-93; miR-126; miR-223; miR-23a; miR-92a | miR-23a; miR-92a; miR-21; miR-100; miR-200b; | miR-320; miR-16; miR-93; miR-126; miR-223 | Exosome | NS | Diagnosis; Prognosis | Liquid biopsy; prognostic factor |
| 8 | Akihiko Yoshimura [72] | 2018 | Original research | miR-99a-5p | miR-99a-5p |  | Exosome | Invasion | Diagnosis; Therapy | Liquid biopsy; Target therapy |
| 9 | Masaki Kobayashi [73] | 2018 | Original research | miR-1290 | miR-1290 |  | Exosome | NS | Diagnosis | Liquid biopsy, Target therapy |
| 10 | Xin Chen [92] | 2018 | Original research | miR-21-3p; miR-125b-5p; miR-181d-5p | miR-21-3p; miR-125b-5p; miR-181d-5p |  | Exosome | regulate the SOCS4/5/STAT3 pathway | Therapy | Target therapy |
| 11 | Wenlong Feng [7] | 2019 | Review | GD3; CD 147; Fas-L; sE-cad; miR-31; miR-214; miR-155; miR-21–3p; miR-125 b-5p; miR-181 d-5p; miR-1246; miR-21; miR-29a; miR-99a-5p; miR-200b; miR-200c; MMP1 | GD3; CD 147; sE-cad; Fas-L; miR-155; miR-21–3p; miR-125 b-5p; miR-181 d-5p; miR-1246; miR-21; miR-29a; miR-99a-5p; miR-99a-5p; MMP1 | miR-31; miR-214; | Exosome | Immunosuppresion; Angiogenesis; CAF Convertion; macrophage polarization; Mesothelial cells' cleavage; Metastasis | Diagnosis; Therapy | Liquid biopsy; Target therapy |
| 12 | Jieru Zhou [3] | 2018 | Original research | hsa-miR-21-5p; hsa-miR-24-3p; hsa-miR-29a-3p; hsa-miR-146b-5p; hsa-miR- 660-5p | hsa-miR-21-5p; hsa-miR-24-3p; hsa-miR-29a-3p; hsa-miR-146b-5p; hsa-miR- 660-5p |  | Exosome | Macrophage polarization; Treg/Th17 ratio; Metastasis | Prognosis | Prognostic factor |
| 13 | Jan Dominik Kuhlmann [100] | 2018 | Original research | hsa-miR-128-3p; hsa-miR- 99a-5p; hsa-let-7i-5p; hsa-miR-148a-3p; hsa-miR-129-5p; hsa-miR-381-3p; hsa-miR-9-3p; hsa-miR-9-5p; hsa-miR- 433-3p; hsa-let-7b-5p; miR-181a; miR-1908; miR-21; miR-486; miR- 223 | hsa-miR-128-3p; hsa-miR- 99a-5p; hsa-let-7i-5p; hsa-miR-148a-3p; hsa-miR-129-5p; hsa-miR-381-3p; hsa-miR-9-3p; hsa-miR-9-5p; hsa-miR- 433-3p; hsa-let-7b-5p; miR-181a; miR-1908; miR-486; miR- 223 |  | Exosome; MV | NS | Platinum resistence | Liquid biopsy; Prognosis factor |
| 14 | Matthias B. Stope [78] | 2017 | Original research | HSP27 | HSP27 |  | Exosome | Substrate for various kinases | Diagnosis | Liquid biopsy |
| 15 | Vanessa A. Enriquez [94] | 2015 | Original research | LIN28A/B; let-7a/b/c/d | LIN28A | let-7a/b/c/d | Exosome | RNA-binding protein that regulates both mRNA and miRNAs | Therapy | NS |
| 16 | Yokoi [7] | 2017 | Original research | MMP1 | MMP1 |  | Exosome; MV | apoptosis in mesothelial cells  leading to peritoneal dissemination | Diagnosis | Liquid biopsy |
| 17 | Barnabas [70] | 2019 | Original research | SERPINB5; S100A14; MYH11; CLCA4; S100A2; IVL; CD109; NNMT; ENPP3 | SERPINB5; S100A14; MYH11; CLCA4; S100A2; IVL; CD109; NNMT; ENPP3 |  | MV | cancer susceptibility gene, regulation of p53, cellular motility | Diagnosis | liquid biopsy |
| 18 | Guerra [99] | 2019 | Original research | RAB 7A |  | RAB 7A | ns | late endocytic pathway, apoptosis, Phagocytosis | Platinum resistence | Target therapy |
| 19 | Zhang [76] | 2019 | Original research | ApoE; EpCAM; Plg; C1q; serpinC1 | ApoE; EpCAM; Plg; C1q | Serpin C1 | Exosome | marker in ovarian cancer and coagulation and complement factors | Diagnosis | Liquid biopsy |
| 20 | Shimizu [81] | 2020 | Review | TGFbeta1, EpCAM, PCNA; TUBB3, EGFR, ApoE, Claudin3, CD147 | TGFbeta1, EpCAM, PCNA; TUBB3, EGFR, ApoE, Claudin3, CD147 |  | Exosome | angiogenesis, immunosuppression, migration, invasion | Diagnosis; Therapy | Liquid biopsy; Target therapy |
| 21 | Nakamura [6] | 2019 | Review | miR21–3p; miR125b-5p; miR181d-5p; miR904; MALAT1; ATF2; MTA1; ROCK1/2; sE-cad; CD44; MMP1; mRNA; miR-99a-5p; miR223;  ARG-1 -; Phosphatidylserine; FasL | miR21–3p; miR125b-5p; miR181d-5p; miR904; MALAT1; ATF2; MTA1; ROCK1/2; sE-cad; CD44; MMP1; mRNA; miR-99a-5p; miR223;  ARG-1 -; Phosphatidylserine; FasL |  | Exosome | angiogenesis, immunosuppression, migration, invasion, markers in ascites | Prognosis; Therapy | Prognostic factor; Immuno thrapy |
| 22 | Yin [9] | 2011 | Original research | annexin A3 | annexin A3 |  | Exosome | exocytosis | Platinum resistence | Target therapy |
| 23 | Kuzmizc [90] | 2019 | Original research | arginase 1 | arginase 1 |  | NS | immunosuppressor(T cell suppressor) | Therapy | Target therapy |
| 24 | Hu [96] | 2017 | Original research | miR-7 | miR7(antitumorale) |  | Exosome | inducer of apoptosis | Therapy | Target therapy |
| 25 | Li [86] | 2017 | Review | miR 21; miR 222; urinary miR 30a5p; miR92a; EPCAM; CD24; ALCAM  Claudin; MMPs; uPA; FasL; miR2223p; TLR; NKG2b; DNAM1 | miR 21; miR 222; urinary miR 30a5p; miR92a; EPCAM; CD24; ALCAM  Claudin; MMPs; uPA; FasL; miR2223p; TLR; NKG2b; DNAM1 |  | Exosome | biomarker in OC; inducer of apoptosis | Diagnosis; Therapy | Prognostic factor; Immuno thrapy |
| 26 | Wang [62] | 2017 | original research | miR-205-5p; miR-145-5p; miR-10a-5p; miR-346, and miR-328-3p | miR-205-5p; miR-145-5p; miR-10a-5p; miR-346, and miR-328-3p |  | Exosome | biomarker in OC | Diagnosis | Liquid biopsy |
| 27 | M.Zhao [80] | 2013 | Original research | HSP27 | HSP27 |  | NS | NS | Diagnosis; Therapeutic issue | Liquid biopsy |
| 28 | Bing Liang [77] | 2012 | Original research | β-actin; EpCAM; hnRNPA1; hnRNPK; Alix | β-actin; EpCAM; hnRNPA1; hnRNPK; Alix |  | NS | NS | Diagnosis; Therapeutic issue | Liquid biopsy |
| 29 | Peng Peng [8] | 2011 | Original research | FasL; TRAIL | FasL; TRAIL |  | NS | NS | Therapeutic issue | Immunomodulation |
